# Pelvic Inflammatory Disease

**DOI:** 10.1155/2011/714289

**Published:** 2012-04-02

**Authors:** Thomas L. Cherpes, Peter A. Rice, Richard L. Sweet

**Affiliations:** ^1^Department of Pediatrics, University of Pittsburgh School of Medicine, Pittsburgh, PA 15224, USA; ^2^Department of Medicine, University of Massachusetts Medical School, Worcester, MA 01605, USA; ^3^Department of Obstetrics and Gynecology, University of California Davis School of Medicine, Sacramento, CA 95817, USA

Pelvic inflammatory disease (PID) is an inflammatory process elicited by the migration of pathogenic microorganisms from the lower to upper genital tract. Although PID is known to increase the risk of tubal factor infertility, many other aspects of this disease remain less well defined. For example, while PID is often caused by *Chlamydia trachomatis* or *Neisseria gonorrhoeae* infection, frequently, neither of these bacterial pathogens is isolated from the upper genital tract of women with PID. This etiologic ambiguity also creates uncertainty regarding the decision to include antibiotics effective against genital mycoplasmas and anaerobic vaginal flora in PID treatment. Moreover, since clinical signs and laboratory measurements do not precisely identify all PID cases, and as the accuracy with which imaging modalities identify upper genital tract inflammation is not firmly established, PID remains a diagnostic challenge. It is clear, however, that better understanding of disease pathogenesis, diagnosis, and treatment is needed to improve the care provided women with PID, and this issue of *Infectious Diseases in Obstetrics and Gynecology* was constructed to deliver specific focus on these topics. 

Three papers in this issue examine the role of *C. trachomatis* in PID pathogenesis. The first examines findings from *C. trachomatis* infection control programs that have altered our understanding of the host immune response to chlamydial infection, and considers implications of these findings for prophylactic vaccine development. The second paper concisely reviews how non-human primate models of chlamydia infection have improved our understanding of PID pathogenesis, while the third explores a possible association between *C. trachomatis*-specific humoral immunity and genital tract inflammation. The fourth PID pathogenesis-focused paper is a case report that reminds readers of the link between *Actinomyces israelii* and this disease among women using intrauterine devices, while the fifth reviews evidence supporting *Mycoplasma genitalium* as a cause of PID.

The five remaining papers in this issue address PID diagnosis and treatment. The first diagnosis-related paper defines a practical approach for the identification of women with PID, while the second tests an algorithm for PID case identification in epidemiological research using administrative diagnostic codes rather than the more unwieldy medical record review. The third describes an investigation among a cohort of women with high prevalence of HIV-1 that found that the conventional markers of histologic endometritis (i.e., neutrophils and plasma cells) are, at least in this population, unreliable surrogate markers for laparoscopically confirmed salpingitis. The fourth paper reviewed existing literature regarding serologic diagnosis of *C. trachomatis* infection in order to construct an algorithm for chlamydial serologic antibody testing in clinical work-up of the infertile couple. We close this issue with a comprehensive review of the antimicrobial therapies currently available for PID treatment.

*Thomas L. Cherpes* *Thomas L. Cherpes* *Peter A. Rice* *Peter A. Rice* *Richard L. Sweet* *Richard L. Sweet*

